# Melanosis intestini: case report

**DOI:** 10.1186/1746-1596-1-3

**Published:** 2006-03-31

**Authors:** Anna Batistatou, John Panelos, Niki J Agnantis

**Affiliations:** 1 Department of Pathology, University of Ioannina Medical School, Ioannina, Greece

## Abstract

The term melanosis in the gastrointestinal tract refers to the accumulation of pigment deposits in the mucosa. Melanosis of the colon is not uncommon and has been associated with certain conditions, however melanosis of the small intestine is extremely rare. Herein, we describe a case in which we observed melanosis not only in the colon, but in the terminal ileum as well, associated with the use of anthraceneline laxatives. The clinical significance of this condition is not clear, however Gastroenterologists and Pathologists should be aware of its existence.

## Background

The term melanosis coli was initially proposed by Virchow [2]. "Melanosis" is a Greek word denoting any condition characterized by abnormal dark coloration of skin or mucosa. Melanosis, by definition, can be due not only to the deposition of melanin, but of other dark-pigmented granules, such as hemosiderin, lipofuskin, lipofuskin-like pigment or ferrum sulfate as well [1,3]. Therefore the recently proposed term "pseudomelanosis", which means a "fake melanosis", and is used for dark colouring of a mucosa due to deposition of pigment other than melanin, is probably not appropriate.

## Case presentation

**Figure 1:**
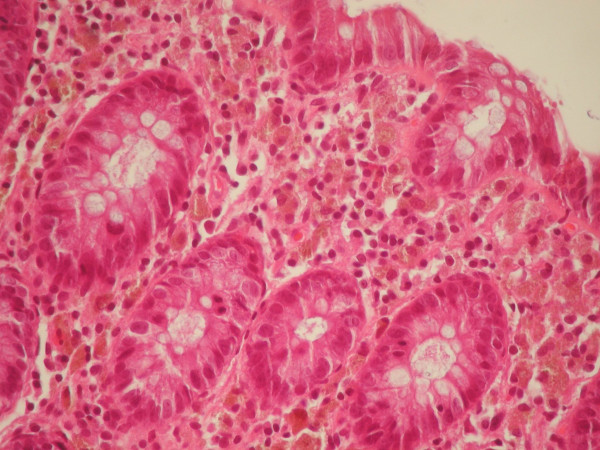
Colonic mucosa. Note the abundant pigment-containing macrophages in the lamina propria (haematoxylin and eosin stain, X400).

**Figure 2:**
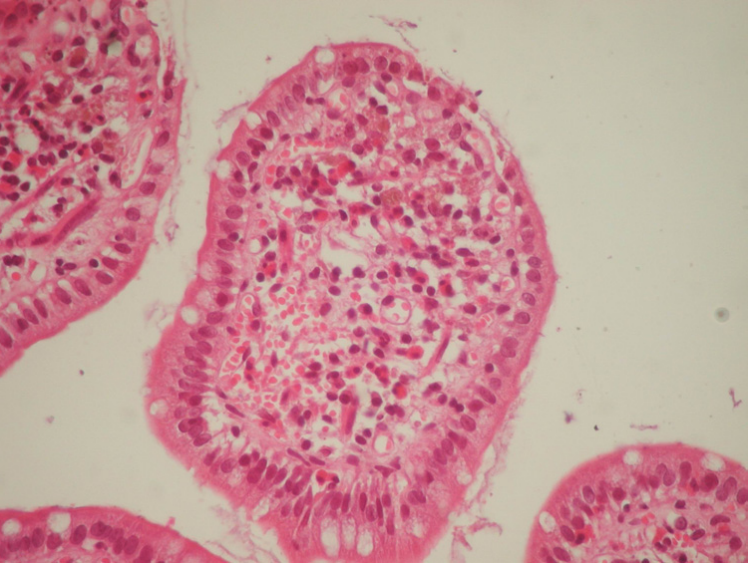
Ileal mucosa. There are fewer, but conspicuous pigment containing macrophages in the lamina propria, particularly at the tips of the villi (haematoxylin and eosin stain, X400).

A 74-year-old man presented complaining of abdominal cramps and bloating. He had a long history of constipation and extensive use of anthraceneline laxatives. Endoscopic examination followed with no remarkable findings and multiple biopsies were taken and sent to the Pathology laboratory for histological evaluation. On microscopic examination, abundant pigment-containing macrophages were noted at the lamina propria of the colon biopsies (Figure 1). Interestingly similar macrophages were seen in the lamina propria of the terminal ileum, particularly at the tips of the villi (Figure 2). Prussian blue stain was negative, indicating that the pigment was not haemosiderin. In addition, the cytoplasm of the pigment-laden macrophages was weakly positive to Periodic Acid-Schiff stain (PAS), but the pigment granules were negative. Based on the histological findings, the diagnosis of melanosis coli was made [3]. A note was added commenting on the appearance of the same findings in the terminal ileum.

## Discussion

Melanosis of the colon is not uncommon and has been associated with the ingestion of anthraceneline laxatives, although it can be observed in patients without such history [3]. Melanosis of the small intestine is an extremely rare finding with only a few cases described in the literature [1,2,4]. Melanosis of the duodenum has been associated with several conditions such as chronic renal failure, gastrointestinal bleeding, ingestion of drugs, or folic acid deficiency [1,2]. There is only one reported case of melanosis of the jejunum, possibly due to ferrous-sulfate administration and vitamin deficiencies [2], and very few cases of melanosis in the ileum [2,4]. In the majority of these cases, the pigment was characterized as hemosiderin and/or lipofuscin. In our case the location and morphological characteristics of the pigment were identical in the ileum and the colon, and presumably were due to the long-term ingestion of anthraceneline. The clinical significance of this condition is not clear, however Gastroenterologists and Pathologists should be aware of its existence.

## Competing interests

The author(s) declare that they have no competing interests.

## Authors' contributions

AB, JP and NJA have been involved in conceiving the manuscript. AB and JP have been involved in the literature search and in drafting the manuscript. All authors read and approved the final manuscript.

## References

[B1] Banai J, Fenyvesi A, Gonda G, Petö I (1997). Melanosis jejuni. Gastrointestinal Endoscopy.

[B2] Ghadially FN, Walley VM (1994). Melanoses of the gastrointestinal tract. Histopathology.

[B3] Rosai J (2004). Rosai and Ackerman's Surgical Pathology.

[B4] Won KH, Ramchand S (1970). Melanosis of the ileum. Am J Dig Dis.

